# Differences in systemic humoral immune response among Balb/c mice administered with probiotic, LPS *Escherichia coli*, and probiotic-LPS *E. coli*

**Published:** 2019-08

**Authors:** Kurniawan Taufiq Kadafi, Satrio Wibowo

**Affiliations:** 1 Department of Pediatrics, Division of Pediatric Emergency and Intensive Care, Saiful Anwar General Hospital, University of Brawijaya, Malang, Indonesia; 2 Department of Pediatrics, Division of Pediatric Gastroenterology and Hepatology, Saiful Anwar General Hospital, University of Brawijaya, Malang, Indonesia

**Keywords:** Humoral, Immune, Probiotic, Lipopolysaccharide

## Abstract

**Background and Objectives:**

The aim of this study was to compare the systemic humoral immune responses, including IgE, IgA, IgG and IgM levels in Balb/c mice administered a probiotic, LPS derived from *Escherichia coli (E.coli)*, and probiotic-LPS derived from *E. coli*.

**Materials and Methods:**

Thirty-two male Balb/c mice, 10–12 weeks of age with body weight ranging from 30–40 g were randomly divided into four experimental groups (n=8). The treatment regimens were as follows: Group 1, mice did not receive LPS or probiotic (control group); Group 2, mice received only LPS on the first day; Group 3, mice received probiotic for 7 days; Group 4, mice received LPS on the first day, and then continued, with probiotic for 7 days. The mice were observed for 8 days, and then, euthanized the next day (day 9). The serum was collected, and the levels of IgE, IgA, IgG and IgM were measured using ELISA.

**Results:**

The humoral immune response was higher in the presence of a probiotic compared to that in the control; IgE (9.02 ± 0.58 units/ml, p=0.000), IgA (3.26 ± 0.99 units/ml, p=0.316), IgG (7.29 ± 0.24 units/ml, p=0.000), and IgM (4.01 ± 2.98 units/ml, p=0.505). When administered with LPS *E. coli* along with probiotic, the humoral immune response was the highest; IgE (10.68 ± 1.63 units/ml, p=0.000), IgA (8.34 ± 1.47 units/ml, p=0.000), IgG (9.96 ± 0.98 units/ml, p=0.000), and IgM (4.31 ± 1.05 units/ml, p=0.319) compared to the control group.

**Conclusion:**

Probiotic-LPS derived from *E. coli* treatment induced a higher humoral immune response (highest IgE, IgA, IgG and IgM levels) compared to treatment with probiotic only.

## INTRODUCTION

Recently, clinical research on the benefits of probiotics has revealed that they can be used to prevent digestive tract infection, reduce the duration of diarrhea, treat *Helicobacter pylori* infection, reduce the risk of cancer, improve mucosal immunity, and prevent allergies (1, 2). The protective effect of probiotics in the gastrointestinal tract is widely known; they act by various mechanisms, including: (i) increasing antimicrobial activity by reducing the pH of intestinal lumen, secreting antimicrobial peptides, inhibiting bacterial infection, inhibiting bacterial adhesion to epithelial cells, (ii) increasing barrier resistance by increasing mucus production (bacteriocin/defensin), (iii) receptor competition, and (iv) modulating the immune system (3). The most commonly used probiotics are *Lactobacillus* and *Bifidobacterium* (4).

In several studies, it was found that probiotics could stimulate innate and adaptive immunity. The effects of probiotics in innate immunity include production of mucin, inhibition of the growth of pathogens, decrease in intestinal permeability, and increase in the activity of natural killer cells, macrophages, and phagocytosis. Reportedly, probiotics induce adaptive immunity by increasing the number of cells that produce IgA, IgG and IgM and increasing the total IgA in the blood and intestinal lumen (4).

Lipopolysaccharide (LPS) is often used as a model of infection of Gram-negative bacteria in experimental studies. Lipopolysaccharide is an endotoxin derived from Gram-negative bacteria. It is a strong inducer of proinflammatory cytokines and stimulates the formation of Th1 cytokines produced by monocytes and macrophages. It could cause severe inflammation of the intestinal mucosa and is the basis for the occurrence of chronic inflammation, such as in inflammatory bowel disease (5–7).

The aim of this study was to investigate the role of probiotic administration on animals that have been exposed to LPS, including the effects of probiotic on humoral immune response, such as the secretion of IgA, IgM, IgG and IgE antibodies.

## MATERIALS AND METHODS

### Probiotic strains, lipopolysaccharide, and administration protocol.

This study used a probiotic preparation (Lacidofil® sachet) containing a culture of *Lactobacillus rhamnosus* R0011, 1.9 × 10^9^ colony-forming units (CFU) and *Lactobacillus achidophillu*s R0052, 0.1 × 10^9^ CFU, with the number of live bacteria (total viable count) being 1.0 × 10^9^ CFU per sachet. The probiotic was administered at a dose of 10^9^ CFU/kgBW/day; thus, each mouse received an average probiotic dose of 3 × 10^8^ CFU. The probiotic was dissolved in 0.4 ml D5% solution. Intragastric administration of probiotic was carried out daily for 7 days. This technique provided a means of accurate dosing of insoluble materials and eliminated the difficulties encountered in the oral administration of suspensions.

LPS from *Escherichia coli* bacteria serotype 055:B5 (Sigma, L2880; Sigma, St.Louis, MO, USA) was administered at a dose of 250 μg/kg BW by dissolving in 0.9% NaCl solution at a dilution ratio of 100:1. Intragastric administration of probiotic was performed on the first day.

### Animal handling and study groups.

Thirty-two male Balb/c mice, 10–12 weeks of age and weighing 30–40 g were purchased from Pusvetma Surabaya, East Java, Indonesia and were kept under the standard conditions. The animals were housed individually in polypropylene cages. The animal room was maintained under hygienic conditions, at a temperature ranging from 22°C to 24°C with a 12-h light/dark cycle and constant humidity over the course of experiment (8 days). The animal received conventional balanced diet (18–20% proteins, 2.5% raw fiber, 5–12% lipid, 60–70% carbohydrates, and vitamins) and water *ad libitum* until the experimental procedures were initiated. All *in vivo* studies were carried out at the Pharmacology Laboratory of Brawijaya University Research Center. This study and animal experiments had been approved by the Health Research Ethics Committee, Faculty of Medicine University of Brawijaya (Ethical clearance approval number: No.249/EC/KEPK-PPDS-JK/10/2011) on the care and use of animals with related codes of practice.

The mice were randomly divided into four experimental groups (n=8 each). The treatment regimens were as follows: Group 1, mice did not receive LPS or probiotic (control group); Group 2, mice received only LPS on the first day; Group 3, mice received probiotic for 7 days; Group 4, mice received LPS on the first day, and then were continued with probiotic for 7 days. The mice were treated and observed for 8 days, and then euthanized the next day (day 9); serum was collected for analyses. The mice were also monitored for the increase in body weight.

### Measurement of the humoral immune responses using ELISA.

Whole blood of mice was collected from the heart after anesthetizing the mice with chloroform. After collection of the whole blood, the blood was allowed to clot by leaving it at room temperature for 15–30 min. Then, the clot was removed by centrifuging at 1,000–2,000 × g for 10 min. Humoral immune responses, including the serum levels of antibodies IgA, IgM, IgG and IgE, were measured by ELISA using a commercially available kit from Immunology Consultant Laboratory, Inc, Oregon, USA with catalog number E-90A, E-90M, E-90G, E-90E, respectively. All ELISA measurements were conducted according to the manufacturer’s instructions.

### Statistical analysis.

The differences between the groups were compared using one way ANOVA followed by a post hoc analysis. Parametric data were presented as mean ± SD, while nonparametric data were presented as median with percentile. Statistical analysis was performed using SPSS software for windows version 16.0 (SPSS, Inc, Chicago, IL). P-value <0.05 was considered significant.

## RESULTS

### Baseline characteristic of each group.

The baseline characteristics of each group are given in Table 1. The body weight of the mice showed no significant changes in the treatment groups. However, the control group showed an increase in body weight.

**Table 1.** Body weight before and after treatment in each group

	**Group 1**	**Group 2**	**Group 3**	**Group 4**
Weight before treatment (g)	34.2 ± 4.47	33.5 ± 3.82	32.75 ± 3.65	30.25 ± 0.71
Weight after treatment (g)	37.25 ± 4.17	33.38 ± 3.93	32.63 ± 2.67	30.88 ± 1.64
P-value	0.017	0.815	0.826	0.180

Values are presented as mean ± standard deviation

P-value was considered significant if P < 0.05

### Serum immunoglobulin (Ig) levels in each group.

**Fig. 1:**
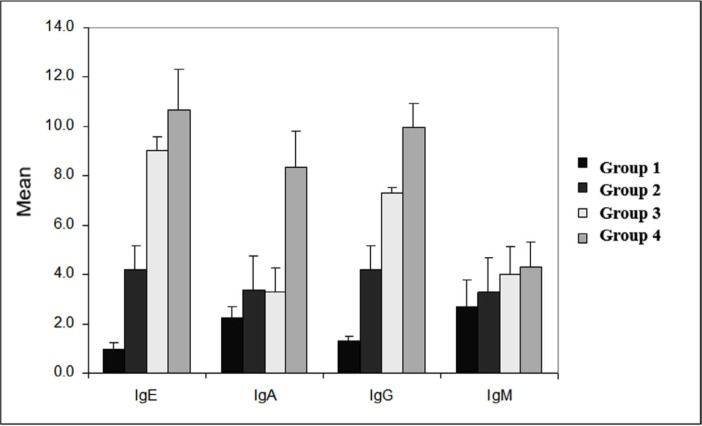
Serum immunoglobulin (Ig) levels in each group Group 1: mice did not receive LPS or probiotic (control group); Group 2: mice received only LPS on the first day; Group 3: mice received probiotic for 7 days; Group 4: mice received LPS on the first day, and then, were continued with probiotic for 7 days. Serum levels of antibodies IgA, IgM, IgG and IgE (unit/mL).

The systemic IgE, IgA, IgG and IgM profiles in mice belonging to the different treatment groups showed varied results (Fig. 1). A strong humoral immune response was elicited in the LPS *E. coli* group, wherein IgE (4.20 ± 0.97 unit/ml), IgA (3.38 ± 1.40 units/ml), IgG (4.22 ± 0.96 units/ml), and IgM (3.27 ± 1.41 unit/ml) levels were higher compared to that in the control group, wherein IgE (0.99 ± 0.26 units/ml), IgA (2.25 ± 0.47 units/ml), IgG (1.31 ± 0.15 units/ml), and IgM (2.73 ± 1.05 unit/ml) (p=0.000, p=0.229, p=0.000, and p=0.932), values were obtained, respectively. The humoral immune response was even more marked in the presence of probiotic, with the levels of IgE (9.02 ± 0.58 units/ml), IgA (3.26 ± 0.99 units/ml), IgG (7.29 ± 0.24 units/ml), and IgM (4.01 ± 2.98 units/ml) being higher compared to the control (p=0.000, p=0.316, p=0.000, and p=0.505, respectively). Thus, a combination of probiotic and LPS *E. coli* elicited the highest humoral immune response, with the levels of IgE (10.68 ± 1.63 units/ml), IgA (8.34 ± 1.47 units/ml), IgG (9.96 ±0.98 units/ml), and IgM (4.31 ± 1.05 units/ml) being the highest compared to that in the control group (p=0.000, p=0.000, p=0.000, and p=0.319, respectively).

## DISCUSSION

Some studies have shown that probiotics could reduce IgE level in the blood and reduce the incidence of allergies through increasing IFN-γ and IL-10. An increase in IFN-γ due to Th1 dominance, and subsequent inhibition of Th2 activation result in a decrease in the IgE levels (8–10). In contrast, in our study, probiotic treatment stimulated an increase in the IgE levels. We found increased blood IgE levels that were significant in the LPS *E. coli*-probiotic group compared to the other groups. It may raise a possibility that LPS *E. coli* itself could stimulate an increase in IgE levels through an increase in Th_2_ cytokines, such as IL_5_. Our results were different compared to those from other studies because we used a different probiotic strain and different research methods. In comparison to those of a previous study, the results of our study were varied due to several factors, such as the strain of probiotics, the type of probiotics (live or dead), duration of probiotics administration, and the concentration of probiotics used. The previous study reported that the efficacy of *Lactobacillus rhamnosus, Steptococcus thermophilus, Lactobacillus delbruecki* subsp. *Bulgaricus* and *Lactobacillus lactis* involved interaction with epithelial cells of the small intestine or interaction with Peyer’s patches, whereas *Lactobacillus acidophilus* worked through interaction with epithelial cells from the large intestine. This result caused different stimulation effects against the humoral immune response, which depends on the probiotics used (12). With the new finding from our research that probiotics could stimulate the increase in IgE levels, especially in the LPS *E. coli* group, it is important that care be taken in clinical applications while using probiotics, especially for the treatment of infectious diseases in patients having atopic disorders.

A study conducted by Pirkka et al. proved that the probiotics of the genus *Lactobacillus* affect the proliferation of B cells and are dependent on the concentration of LPS. The use of *Lactobacillus acidophilus* has been shown to increase the proliferation of B cells to 43% and improved the response to LPS. The opposite results were obtained in probiotics *Lactobacillus casei, Lactobacillus gasseri* and *Lactobacillus rhamnosus*, which inhibited the proliferation and mitogenic stimulation of lymphoproliferation, as well as inhibited the achievement of the optimal concentration of LPS. Pirkka et al. concluded that the effects of immune responses from probiotics could not be extrapolated to other probiotics, even though the probiotcs came from the same genus. Therefore, we also have to be careful in using probiotics, especially during the immunosuppressive states (13).

In our study, we found that IgA levels in the blood increased slightly in the LPS *E. coli*-probiotic group compared to the other groups. Another study showed no significant difference between the group administered with LPS *E. coli* and the group administered only probiotic in increasing the IgA levels, which could be because probiotics were not able to generate humoral immune responses (14). A study reported that after the administration of probiotics for 2 days, fractions of peptides from the probiotics could increase the number of B cells in the lamina propria; the administration of probiotics for 5 days increased the number of B lymphocytes in the intestine, and probiotics administration for 10 days increased the number of B lymphocytes and IgA in the intestine and blood (15). Thus in our study, IgA levels in the blood were measured on the 8^th^ day after administering probiotic for 7 days. We had hypothesized that probiotics could increase IgA secretion in the intestine but would not be able to generate systemic IgA in blood.

Few studies showed that the administration of LPS *E. coli* and probiotics could increase IgG level in the blood. A research conducted by Heras et al. in rats proved that administering LPS *E. coli*-probiotic (*Lactobacillus plantaris*) induced higher IgG levels in the blood compared to in the control; the IgG level started increasing in the first week, and then, increased significantly in the 5^th^ week (16). Our study also proved that after 7 days of probiotic administration, the groups administered with LPS *E. coli* and LPS *E. coli*-probiotic showed a significant increase in the IgG level compared to the control group. It has been reported that administering LPS *E. coli* and probiotic in the form of *Bifidobacterium bifidum* (10^4^ bacteria/ml) could increase the serum IgA and IgG levels compared to in the control group. In addition, *Bifidobacterium* has a direct mitogenic effect on the B lymphocytes, which could modulate antibody responses; thus, using it in combination with LPS *E. coli* was profitable because LPS *E. coli* could act as an activator of polyclonal B lymphocytes (17).

Based on the findings of our study, we conclude that we should apply probiotics in clinical medicine, keeping in mind certain complication factors; for example, while using probiotics for the treatment of acute diarrhea in children, we must pay attention to the atopy factors that exist in these patients. If the patient already has a family history of atopy, probiotic therapy could possibly stimulate allergies. Diarrhea in children also causes allergy to cow milk proteins; therefore, we should not administer probiotics to those children because it will trigger severe diarrhea.
